#  Pneumothorax in a Neonate after Foreign Body Ingestion 

**Published:** 2016-04-10

**Authors:** Prashant Sadashiv Patil, Paras Kothari, Abhaya Gupta, Geeta Kekre, Vishesh Dikshit, Ravi Kamble, Kedar Mudkhedkar

**Affiliations:** Department of Pediatric Surgery, Lokmanya Tilak Municipal Medical College and General hospital, Mumbai, India

**Dear Sir**

A 5-day-old male neonate was referred for removal of foreign body. Baby had accidentally ingested a gold finger ring presented to her by grandmother. Baby was brought to outside hospital in stable clinical condition. Initial X-ray neck and chest showed radio-opaque foreign body at level of cricopharynx. Baby was posted for rigid esophagoscopy under anesthesia. But foreign body was not found in the esophagus. X-ray was repeated on table under C-ARM guidance which showed foreign body in stomach. Due to non-availability of flexible endoscope baby was referred to our hospital. Baby was found to have tachypnea on admission. Repeat chest X-ray showed pneumothorax on right side along with radio-opaque foreign body in stomach (Fig. 1). Intercostal tube was put and baby was kept under close observation. Baby improved symptomatically. Definite cause for pneumothorax could not be found. Perforation at level of cricopharynx due to rigid endoscopy may be postulated. Baby was kept nil by mouth for 6 days and oral feeds were gradually started. Baby passed ring spontaneously on 7th day of hospital admission. 

Foreign bodies are frequently swallowed by accident, during play. Foreign body in the esophagus of the neonate may produce respiratory symptoms, due to the compression on the tracheal wall. Marked respiratory symptoms were described in a 19-day-old neonate, with a metal key in his esophagus [1, 2]. The foreign body ingestion could lead to life-threatening symptoms, such as the reported intestinal obstruction in a 4-day-old neonate who swallowed a grape [3]. Fortunately most foreign bodies pass spontaneously without any complication. Foreign bodied that are too large to pass pylorus need removal by flexible endoscope. If the incident has not been witnessed and the ingested object is radiolucent, the diagnosis of foreign body ingestion can be very tricky in neonates. Beside typical symptoms of choking, gagging, dysphagia or drooling, esophageal foreign body in neonates has been associated with hematemesis and malena.[4, 5] A simple x-ray followed by a contrast study (if needed) can help in establishing the diagnosis. Fortunately in our cases the diagnosis was not much of a problem as one had a radio opaque foreign body (finger ring) evident on x-ray.

**Figure:**
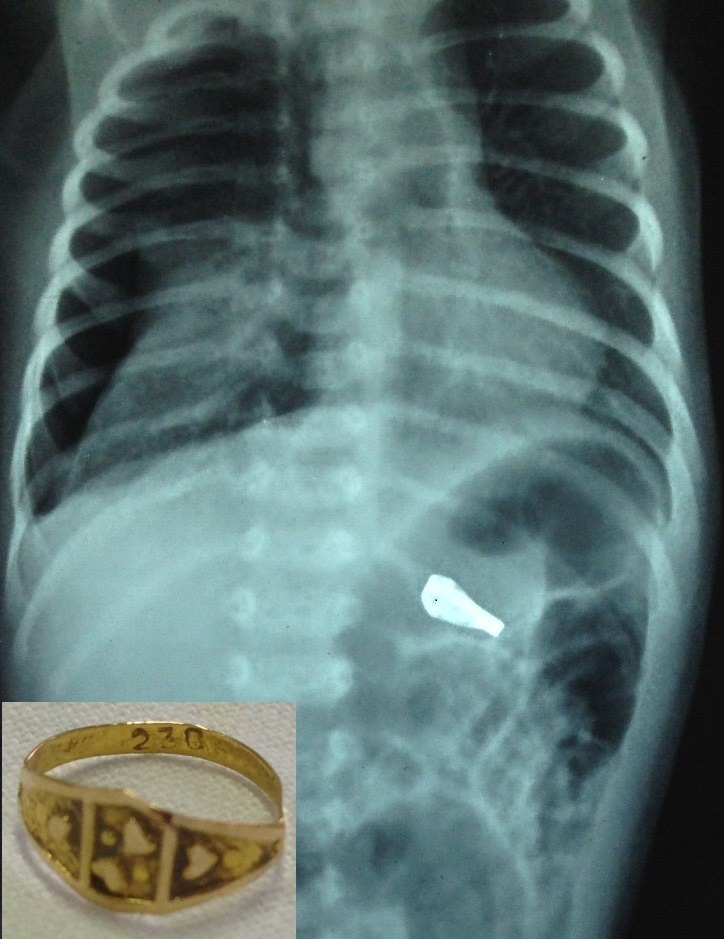
Figure1: Right side pneumothorax along with Radio-opaque foreign body in stomach. Inset shows retrieved ring.

## Footnotes

**Source of Support:** Nil

**Conflict of Interest:** None

## References

[1] Chmielik LP, Frąckiewicz M, Chmielik M. Diagnostic and therapeutic difficulties in cases of foreign body in the oesophagus in children treated in the paediatric end clinic WUM in 2005–2009. Case Rep. New Medicine. 2009; 4:85–8.

[2] Sanjeeb S, Bora P. Key as unusual esophageal foreign body in neonate. Ped Rad (serial online) 2009; 9(4) www.PedRad.info/?20090420160623 (accessed 13.01.2010)

[3] Tander B, Baskin D, Mutlu G, Sever N, Bulut M. An unusual foreign body in the bowel lumen causing obstruction in neonate. J Pediatr Surg. 1999; 34:1289–90. 10.1016/s0022-3468(99)90173-2 10466617

[4] Chowdhury CR, Bricknell MCM, MacIver D. Oesophageal foreign body: an unusual cause of respiratory symptoms in a three-week-old baby. J Laryngol Otol. 1992; 106:556-7. 10.1017/s0022215100120134 1624897

[5] Thapa BR, Kaur B, Nagi B, Dilawari JB. Unusual foreign body (stone) in the esophagus of a neonate mimicking tracheoesophageal fistula. Indian Pediatr. 1993; 30:943-5. 8132295

